# Piecing it together: atrophy profiles of hippocampal subfields relate to cognitive impairment along the Alzheimer’s disease spectrum

**DOI:** 10.3389/fnagi.2023.1212197

**Published:** 2023-10-31

**Authors:** Nicholas J. Christopher-Hayes, Christine M. Embury, Alex I. Wiesman, Pamela E. May, Mikki Schantell, Craig M. Johnson, Sara L. Wolfson, Daniel L. Murman, Tony W. Wilson

**Affiliations:** ^1^Institute for Human Neuroscience, Boys Town National Research Hospital, Boys Town, NE, United States; ^2^Center for Mind and Brain, University of California, Davis, CA, United States; ^3^Department of Psychology, University of Nebraska at Omaha, Omaha, NE, United States; ^4^Montreal Neurological Institute, McGill University, Montréal, QC, Canada; ^5^Department of Neurological Sciences, University of Nebraska Medical Center, Omaha, NE, United States; ^6^College of Medicine, UNMC, Omaha, NE, United States; ^7^Department of Radiology, UNMC, Omaha, NE, United States; ^8^Geriatrics Medicine Clinic, UNMC, Omaha, NE, United States; ^9^Memory Disorders and Behavioral Neurology Program, UNMC, Omaha, NE, United States; ^10^Department of Pharmacology and Neuroscience, Creighton University, Omaha, NE, United States

**Keywords:** dementia, medial temporal lobe, neurodegeneration, neuropsychology, biomarkers

## Abstract

**Introduction:**

People with Alzheimer’s disease (AD) experience more rapid declines in their ability to form hippocampal-dependent memories than cognitively normal healthy adults. Degeneration of the whole hippocampal formation has previously been found to covary with declines in learning and memory, but the associations between subfield-specific hippocampal neurodegeneration and cognitive impairments are not well characterized in AD. To improve prognostic procedures, it is critical to establish in which hippocampal subfields atrophy relates to domain-specific cognitive declines among people along the AD spectrum. In this study, we examine high-resolution structural magnetic resonance imaging (MRI) of the medial temporal lobe and extensive neuropsychological data from 29 amyloid-positive people on the AD spectrum and 17 demographically-matched amyloid-negative healthy controls.

**Methods:**

Participants completed a battery of neuropsychological exams including select tests of immediate recollection, delayed recollection, and general cognitive status (i.e., performance on the Mini-Mental State Examination [MMSE] and Montreal Cognitive Assessment [MoCA]). Hippocampal subfield volumes (CA1, CA2, CA3, dentate gyrus, and subiculum) were measured using a dedicated MRI slab sequence targeting the medial temporal lobe and used to compute distance metrics to quantify AD spectrum-specific atrophic patterns and their impact on cognitive outcomes.

**Results:**

Our results replicate prior studies showing that CA1, dentate gyrus, and subiculum hippocampal subfield volumes were significantly reduced in AD spectrum participants compared to amyloid-negative controls, whereas CA2 and CA3 did not exhibit such patterns of atrophy. Moreover, degeneration of the subiculum along the AD spectrum was linked to a significant decline in general cognitive status measured by the MMSE, while degeneration scores of the CA1 and dentate gyrus were more widely associated with declines on the MMSE and tests of learning and memory.

**Discussion:**

These findings provide evidence that subfield-specific patterns of hippocampal degeneration, in combination with cognitive assessments, may constitute a sensitive prognostic approach and could be used to better track disease trajectories among individuals on the AD spectrum.

## 1. Introduction

The human medial temporal lobe serves as the anchor to our episodic memories; binding together environmental features into declarative relationships (Tulving, 1972; Eichenbaum and Cohen, 2004; Schiller et al., 2015; Eichenbaum, 2017a, b). While normal aging is often accompanied by the steady decline of the ability to form these memories, people with probable Alzheimer’s disease (AD) experience such declines much more rapidly (Schuff et al., 2008; Kurth et al., 2017; Jack et al., 2018; McKeever et al., 2020). A myriad of studies have shown that the degeneration of the medial temporal lobe in people on the AD spectrum [ADS; i.e., amyloid-positive individuals with AD or mild cognitive impairment (MCI)], and in particular atrophy of the hippocampal formation, predicts cognitive decline in these individuals, and thus represents a clear target for further investigation (Braak and Braak, 1991; Mueller et al., 2005, 2010; Apostolova et al., 2006; Schuff et al., 2008; Seeley et al., 2009; Frisoni et al., 2010; La Joie et al., 2013; Sarica et al., 2018). However, the specificity of hippocampal volumetrics for predicting eventual cognitive decline has been less than desirable (Mueller et al., 2005, 2010, 2018; Wolk et al., 2017), particularly at earlier stages of AD (La Joie et al., 2013; Wolk et al., 2017; Mueller et al., 2018). Thus, more refined interrogations of hippocampal degeneration patterns and their association to impaired cognitive functioning in ADS are necessary to advance our understanding of the disease and the role of the hippocampus in normative cognitive function.

Recent studies suggest the likely culprit for this low predictive specificity is the complex makeup of the hippocampal formation (Mueller et al., 2018). The hippocampus is comprised of histologically heterogeneous and genetically distinguishable subfields (Duvernoy, 2005; Patel et al., 2017; van der Meer et al., 2020). For example, recent literature has demonstrated the predictive power of family history and polygenic risks of AD for atrophy in specific subfields, such that CA1 and subiculum subfield volumes are significantly decreased among individuals with increased genetic risk of AD (Mueller et al., 2008; Schuff et al., 2008; O’Dwyer et al., 2012; Tardif et al., 2018; McKeever et al., 2020; Murray et al., 2021). Moreover, this degeneration of the CA1 and subiculum subfields is evident in people with MCI (Apostolova et al., 2006, 2010; Mueller et al., 2010, 2018; Hanseeuw et al., 2011; Carmichael et al., 2012; La Joie et al., 2013; Khan N. A. et al., 2015; Yushkevich et al., 2015; Kälin et al., 2017), as well as in AD (Apostolova et al., 2010; Mueller et al., 2010; Carmichael et al., 2012; La Joie et al., 2013; Wisse et al., 2014; Khan W. et al., 2015; Wolk et al., 2017; Huang et al., 2020; Izzo et al., 2020). Although there are volumetric changes to the whole hippocampus (Apostolova et al., 2012; Thomann et al., 2013) and to its subfields (Apostolova et al., 2012; Kurth et al., 2017; Malykhin et al., 2017; Parker et al., 2019) associated with aging in the absence of any neurodegenerative disease, hippocampal subfield changes observed in AD have been linked to clinical symptom progression (Atienza et al., 2011; Fouquet et al., 2012; Carlesimo et al., 2015; Broadhouse et al., 2019; Ogawa et al., 2019; Zhao et al., 2019; Nurdal et al., 2020). Importantly, these studies have primarily assessed within-group associations between hippocampal volumetrics and cognitive assessments in AD, without controlling for normal age-related declines in hippocampal size. Accordingly, these methods do not allow for strong inferences regarding the specific impact of AD-related subfield degeneration on cognitive outcomes (Jack et al., 2002; La Joie et al., 2013).

The main goal of the present study was to examine how AD alters hippocampal subfield profiles using state-of-the-art methods both in the acquisition of structural scans and in segmentation of the structure. Moreover, this study sought to unravel how these pathological changes are associated with cognitive functioning as measured by standard neuropsychological tools. In line with the amyloid, tau, neurodegeneration framework (ATN; Jack et al., 2018) for AD research, all participants were biomarker confirmed for amyloid positivity/negativity status. High-resolution structural segmentations of hippocampal subfields from 29 biomarker-positive people on the ADS and 17 demographically matched biomarker-negative, cognitively-normal older adults were analyzed to assess levels of degeneration in the hippocampal subfields. We then modeled these data alongside neuropsychological measures of learning, memory, and general cognitive status to determine how profiles of hippocampal subfield degeneration relate to performance on clinically-relevant cognitive measures. We hypothesized that associations between specific subfields (e.g., CA1) and cognitive markers (e.g., memory) would emerge, informing which patterns of neurodegeneration in the human hippocampus are linked to cognition, thereby further bridging the gap between biological markers of pathology and cognitive impairment across the ADS.

## 2. Materials and methods

### 2.1. Participants

All participants were recruited from the greater Omaha area as part of a multimodal imaging study of neural dynamics in AD (the dynamic mapping of Alzheimer’s disease pathology [DMAP] study; see Wiesman et al., 2021, 2022). This investigation was reviewed and approved by the Institutional Review Board at the University of Nebraska Medical Center. A detailed description of the study was provided to all participants, including informants for participants on the ADS. Individuals on the ADS whose capacity to consent was questionable provided written informed assent accompanied by written consent from a legally-authorized representative. All other participants provided written informed consent. Informants for each participant in the ADS group (regardless of capacity) were also consented for collection of additional data and completion of questionnaires.

Forty-four participants (*n* = 44) with amnestic MCI (aMCI) or mild probable AD, and a group of cognitively-normal healthy older adults (*n* = 20) were enrolled in this study. Participants with amnestic complaints were recruited from local Memory Disorders and Geriatrics Clinics and categorized as aMCI or probable AD by a fellowship-trained neurologist specializing in memory disorders. To confirm biomarker status, participants underwent amyloid PET imaging. All scans were read by a fellowship-trained neuroradiologist blinded to group assignment and assessed as being “amyloid-positive” or “amyloid-negative” using established clinical criteria. For all participants, exclusion criteria included neurological or psychiatric disorders (other than aMCI/AD), history of head trauma, moderate or severe depression (Geriatric Depression Scale ≥10), current substance abuse, or incomplete data.

Data from 18 participants were excluded due to the following: COVID-related withdrawal (ADS, *n* = 1), major incidental finding (ADS, *n* = 1), amyloid-negativity in cognitively impaired group (*n* = 4), MRI (T1 or TSE) scan quality (ADS, *n* = 9; Controls, *n* = 2; see below for more detail), no amyloid status (Controls, *n* = 1). The final sample for ADS included 29 amyloid-positive participants (aMCI = 12; AD = 17), and 17 amyloid-negative for the cognitively normal control group.

### 2.2. Neuropsychological assessments

The neuropsychological data used in the present study is similar to previous reports (Wiesman et al., 2021, 2022). Briefly, participants completed the Functional Activities Questionnaire (FAQ), the Montreal Cognitive Assessment (MoCA) and the Mini-mental State Examination (MMSE; Folstein et al., 1975; Pfeffer et al., 1982; Nasreddine et al., 2005). All participants also underwent a battery of neuropsychological tests that assessed the following domains: attention and executive function (Wechsler Adult Intelligence Scale [WAIS-IV] Digit Span Forward, Backward, and Sequencing; Trail Making Test Part B), language (Boston Naming Test; Controlled Oral Word Association Test/Phonemic Verbal Fluency; Animals/Semantic Verbal Fluency), processing speed (WAIS-IV Digit Symbol Coding; Trail Making Test Part A; Heaton et al., 2004; Wechsler, 2008), immediate recollection (Wechsler Memory Scale Fourth Edition [WMS-IV] Logical Memory I Immediate Recall; Hopkins Verbal Learning Test-Revised [HVLT-R] Learning Trials 1–3), and delayed recollection (WMS-IV Logical Memory II Delayed Recall and Recognition; HVLT-R Delayed Recall and Recognition Discriminability Index; Brandt and Benedict, 2001; Wechsler, 2008).

Raw scores for each participant were converted to demographically-adjusted z-scores (e.g., based on age, education, etc.) using published normative data and following standard practice procedures (Brandt and Benedict, 2001; Heaton et al., 2004; Wechsler, 2008, 2009). Demographically corrected z-scores based on test-specific normative data were then averaged across tests to create composite cognitive domain z-scores by participant. From this battery, the neuropsychological tests which have been shown to measure processes commonly attributed to hippocampal functions (i.e., immediate and delayed recollection; Atienza et al., 2011; Fouquet et al., 2012; Carlesimo et al., 2015; Broadhouse et al., 2019; Ogawa et al., 2019; Zhao et al., 2019; Nurdal et al., 2020) and those which assess general cognitive status (i.e., MMSE and MoCA) were considered in statistical analyses. Note that immediate and delayed recollection were modeled as separate cognitive domains: while they share considerable variance, immediate vs. delayed learning and memory represent functions with important conceptual distinctions. Descriptive statistics for all neuropsychological measures are reported in Table 1.

**Table 1:** Demographic, neuropsychological, and neurological summary.

	ADS (aMCI = 12; AD = 17)	Controls (17)	est.	2.5%	97.5%	*p*-value
Demographics						
Age (yrs)	70 _(6.12 [57,84])_	73 _(3.71 [70,84])_	1.83	−0.31	6.31	0.07
Education (yrs)	15.10 _(2.69 [10,20])_	16.24 _(2.86 [12,20])_	1.32	−0.61	2.87	0.19
Sex (f/m) †	14/15	6/11	0.59	−0.50	0.17	0.44
Neuropsychology						
MMSE	23.66 _(4.30 [16,30])_	29.18 _(1.13 [26,30])_	6.55	3.80	7.24	< 0.001 *
Immediate recollection	−2.12 _(0.82 [−3.05,-0.13])_	0.56 _(0.73 [−0.82,1.82])_	11.40	2.19	3.15	< 0.001 *
Delayed recollection ††	−2.25 _(0.76 [−2.85,0.57])_	0.26 _(0.55 [−1.04,0.99])_	480	2.34	3.01	< 0.001 *
MoCA	18.83 _(5.10 [11,26])_	27.50 _(1.73 [25,30])_	8.09	6.51	10.84	< 0.001 *
Verbal fluency	−1.13 _(0.99 [−2.97,0.77])_	0.23 _(0.80 [−1.13,1.60])_	4.67	0.71	1.79	< 0.001 *
Processing speed	−1.01 _(1.32 [−3.23,1.47])_	0.77 _(0.82 [−0.77,2.50])_	5.65	1.15	2.42	< 0.001 *
Attention and executive function	−0.94 _(0.99 [−2.84,1.31])_	0.55 _(0.57 [−0.73,1.35])_	6.48	1.03	1.95	< 0.001 *
Brain structure						
Whole Hippocampal Volume (mm^3)	922 _(395 [−118,1,649])_	1,455 _(257 [821,1910])_	5.53	338	727	< 0.001 *
Global Cortical Thickness (mm)	2.40 _(0.39 [1.54,4.21])_	2.55 _(0.44 [1.88,4.15])_	4.07	0.08	0.23	< 0.001 *

Neuropsychological composite z-scores. MMSE and MoCA scored /30. All hypothesis tests used an independent samples *t*-test unless annotated otherwise. Variable means are listed with standard deviations shown in parentheses and ranges in brackets. Raw counts for sex are listed. aMCI, amnestic Mild Cognitive Impairment; ADS, Alzheimer’s Disease Spectrum; MMSE, Mini-Mental State Exam. † = Chi-square test, †† = Wilcoxon test. *p < 0.001.

### 2.3. Florbetapir PET data acquisition and processing

The florbetapir-PET data acquisition and preprocessing in the present study was similar to previous reports (Wiesman et al., 2021, 2022). All scans were read by a fellowship-trained neuroradiologist blinded to group assignment and assessed as being “amyloid-positive” or “amyloid-negative” using established clinical criteria. Briefly, 18F-florbetapir (Amyvid^™^, Eli Lilly) PET data were collected on a GE Discovery MI digital scanner (Waukesha, WI), reconstructed, body-weight normalized, and MNI-normalized in MIMNeuro (Joshi et al., 2012; Minoshima et al., 2016). Data were then normalized to the crus of the cerebellum (SUIT template), back-transformed into each patient’s native structural MRI space (mri_vol2vol), smoothed to a common resolution (8 mm FWHM), projected onto native surfaces (mri_vol2surf; maximum value; projection fraction = 1; steps of 2), and then normalized to FSAverage template surface using FreeSurfer (Diedrichsen, 2006; Fischl, 2012; Landau et al., 2012; Guo et al., 2020). Surface masks from seven target regions of interest of the Desikan-Killiany Atlas (parahippocampal, entorhinal, inferior and superior parietal, precuneus, lateral occipital, and superior frontal; Joshi et al., 2012; Landau et al., 2012) were used to compute mean standardized uptake value ratios (SUVr) of amyloid deposition per patient on the ADS.

### 2.4. MRI data acquisition and processing

During data acquisition, participants were in constant contact with research personnel through real-time audiovisual monitoring. Structural MRI data were collected on a 3 T Siemens Prisma System with a 64-channel head coil, and included a medial temporal lobe focused T2-weighted turbo spin echo (TSE) image [TR = 7.79 s, TE = 66 ms, flip angle = 145°, FOV = 170 mm, in plane resolution = 0.4 × 0.4 mm, slice thickness = 2 mm, slices = 32] and a whole-head T1-weighted three-dimensional 1 mm isotropic MPRAGE sequence [TR = 2.3 s, TE = 2.98 ms, flip angle = 9°, FOV = 256 mm], per best modern practices (Yushkevich et al., 2015; Mueller et al., 2018; Olsen et al., 2019; Wisse et al., 2021).

**Figure 1:**
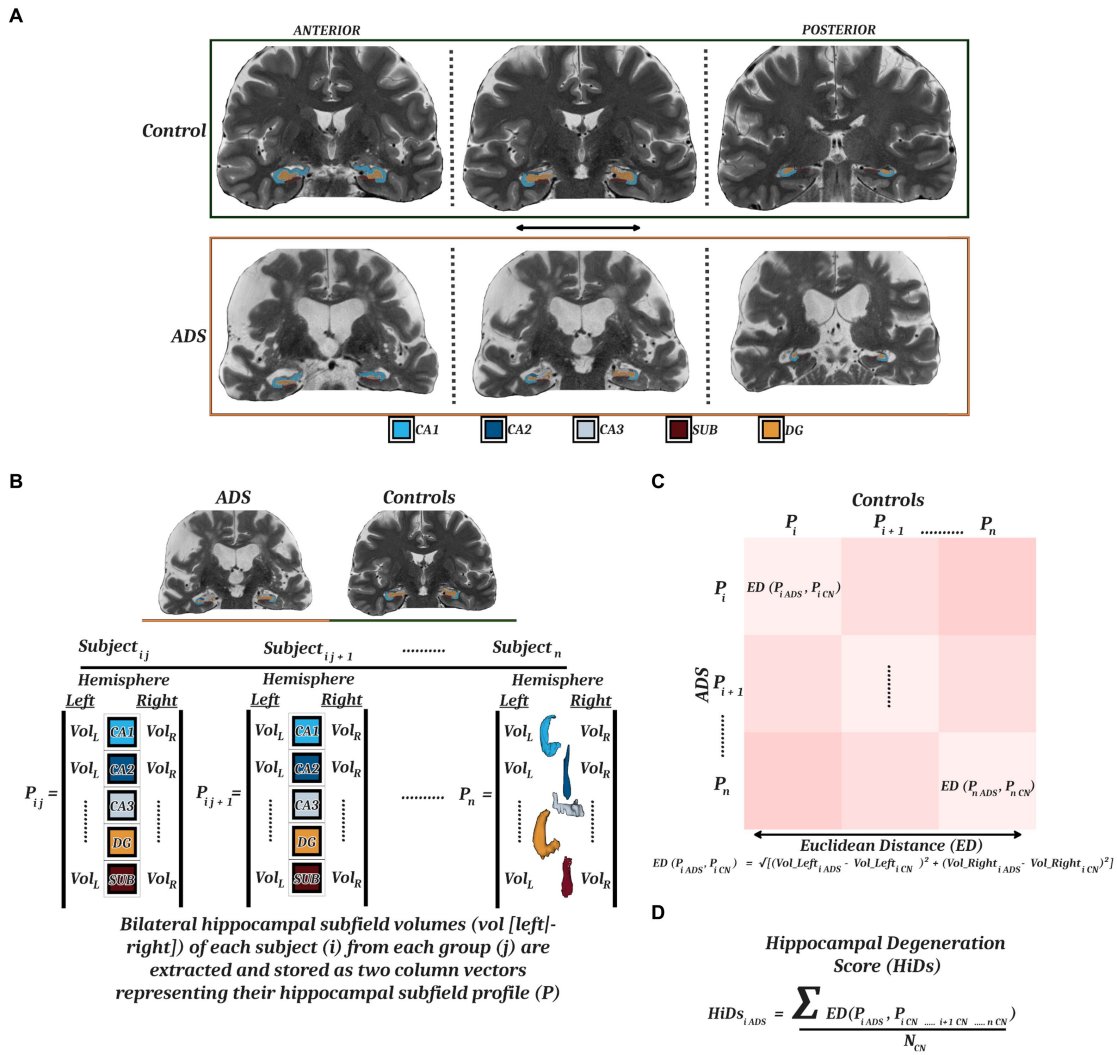
Hippocampal parcellation and computation of degeneration scores. **(A)** MRI T2-weighted images from a representative control (top) and participant on the ADS (bottom) with superimposed ASHS parcellations of five hippocampal subfields (CA1, CA2, CA3, DG, SUB) along the anterior–posterior axis. **(B)** ICV-adjusted bilateral hippocampal subfield volumes were extracted to generate individual hippocampal subfield profiles for each participant. These profiles were vectorized and used to compute a Euclidean distance matrix **(C)** representing the multidimensional dissimilarity between each patient-control pairing. **(D)** By taking the mean of the Euclidean distances from all healthy controls for each participant on the ADS, we derived hippocampal degeneration scores (HiDs) representing the degree of AD-specific hippocampal atrophy. MRI, magnetic resonance imaging; ASHS, Automated Segmentation of Hippocampal Subfields; CA, cornu ammonis; DG, dentate gyrus; SUB, subiculum; ADS, Alzheimer’s Disease Spectrum.

MRI Quality Control (MRIQC) v0.16.1 (Esteban et al., 2017) was used as an initial check of MRI data quality, and these preliminary automated ratings were supplemented with systematic quality assessment by a trained rater. Assessment included a review of hippocampal coverage/completeness, contrast/noise, and motion artifacts. Data from eight participants were excluded due to motion artifacts in the T2 TSE images. MRI scans that survived quality assessment of the raw data were then processed through the segmentation workflow. High resolution hippocampal subfield volumes were quantified with the Automatic Segmentation of Hippocampal Subfields (ASHS) software and the UPENN atlas consisting of scans of MCI individuals and older adults (Yushkevich et al., 2015). Briefly, the dedicated T2-weighted image of the hippocampi was coregistered to the routine T1-weighted image, segmented, and bias-corrected. Next, hippocampal subfields were parceled from the medial temporal lobe via multi-atlas joint-label fusion with multiple pairwise registrations, consensus segmentation, learning-based error correction, and bootstrapping. This method was chosen as it has been shown to perform with similar or better accuracy as compared to other methods employed for work with similar clinical populations (de Flores et al., 2015; Yushkevich et al., 2015; Giuliano et al., 2017; Mueller et al., 2018; Olsen et al., 2019). From the resulting parcellation, we used the following volumes for our analyses: CA1, CA2, CA3, dentate gyrus, and subiculum (Figure 1A). Data from 3 participants were excluded at this stage due to segmentation errors. All hippocampal ROI’s were reviewed by a trained rater for coverage/completeness. As a result, 46 (ADS = 29, Controls = 17) images survived quality control and/or segmentation procedures, and were used for all subsequent analyses, and 11 (ADS = 9, Controls = 2) images were excluded. Importantly, participants included in our final sample after imaging-based exclusion did not significantly differ in terms of demographics (i.e., Age, Education, Sex) or cognition (i.e., MoCA, MMSE) from participants who were excluded at this MRI processing stage (Supplementary Table S1). T1-weighted images for all subjects that passed quality control were also processed through the recon-all workflow of FreeSurfer (v7.1.1; Fischl, 2012) which produced segmentations of whole-hippocampal volumes and derivations of global cortical thickness. These whole-hippocampal segmentations were reviewed by a trained rater following the same procedures as described above for subfields.

### 2.5. Statistical analyses

To estimate the extent of disease-related atrophy and to control for inter-individual variation in brain size, individualized intracranial volume estimates (ICV) were regressed from the hippocampal subfield volume estimates resulting in ICV-adjusted hippocampal subfield volumes (Buckner et al., 2004; Raz et al., 2004; Voevodskaya et al., 2014; Pintzka et al., 2015). Moreover, all analyses considered age, sex, and education as potential covariates to minimize any potential confounding effects on outcome measures (Mueller and Weiner, 2009; La Joie et al., 2010; Piras et al., 2011; Apostolova et al., 2012; Noble et al., 2012; Farfel et al., 2013; Malykhin et al., 2017; Tang et al., 2017; O’Shea et al., 2018; van Eijk et al., 2020; Veldsman et al., 2021). Inclusion of these potential additional covariates (i.e., age, sex, education) was determined per each model by comparing models with successive inclusion of potential covariates (i.e., model 1: age, model 2: age + sex, model 3: age + sex + education) and selecting the model that was best at explaining outcome variance, using the *anova* function in R. Models including covariates that significantly improved prediction vs. the no-covariate base model (*p* < 0.05) were selected. Models that included any of these additional potential covariates are listed in Supplementary Table S2. All models were also inspected for normality with plots of the residuals against fitted values and using the Shapiro–Wilk test. When the assumption of normality of residuals was violated, instead of parametric linear models we employed a nonparametric Wilcoxon test or nonparametric permutation testing to derive inferential statistics (*wilcox.test* and *lmPerm* in R). The models that required nonparametric tests are indicated in Supplementary Table S2.

We first computed a series of general linear models to examine any group differences in whole hippocampal and hippocampal subfield volumes. Next, we sought to determine how degeneration of the hippocampus relates to impairments in immediate and delayed recollection, and general cognitive status, in participants on the ADS. To quantify hippocampal degeneration in participants on the ADS relative to controls, we first created hippocampal volume profiles by vectorizing the left and right hemisphere volumes for each subfield for each participant. We then computed a Euclidean distance matrix between all combinations of patient (N) and control (M; Figure 1B), resulting in an NxM matrix of distance metrics that represents the dissimilarity between each ADS participant’s subfield profile and every control’s subfield profile (Figure 1C). This multidimensional approach is preferable to simpler metrics in that it allows for the simultaneous consideration of several subfields from right and left hemispheric volumes to ensure asymmetric hemispheric variability is also captured (Maruszak and Thuret, 2014; Sarica et al., 2018; Ogawa et al., 2019; Zhao et al., 2019; Huang et al., 2020). For the analyses in this study, these Euclidean distance values were averaged across all controls per each participant on the ADS, thereby representing the mean dissimilarity of each ADS participant hippocampal subfield profile from controls across hemispheres, termed hippocampal degeneration score (HiDs; Figure 1D). We then used these HiDs to test how decrements across hemispheres were associated with cognitive domain scores using partial correlations. We focused these analyses on the MMSE and MoCA, and the composite domain scores representing learning and memory as prior studies have found that these cognitive measures are most robustly linked to hippocampal volumes (Atienza et al., 2011; Fouquet et al., 2012; Carlesimo et al., 2015; Broadhouse et al., 2019; Ogawa et al., 2019; Zhao et al., 2019; Nurdal et al., 2020).

Extant research suggests there is more to be gained than lost by demarcating the hippocampus into subregions. For example, Wolk et al. (2017) have argued that global hippocampal measures obscure critical information at early stages of disease progression, and others have shown measures of subfields outperform whole hippocampus measures to discriminate controls from individuals on the ADS (Mueller et al., 2010; Maruszak and Thuret, 2014). To understand whether the degeneration of individual subfields was associated with cognitive impairments, we followed the same HiD procedure as above to quantify degeneration, but separately for each respective subfield using the vectorized left and right hemisphere values. Moreover, to determine whether any of the reported associations between degeneration of subfields and cognition could be explained by non-specific cortical tissue degeneration, we also computed models substituting global cortical thickness estimates from FreeSurfer (averaged over all cortical locations per participant) in the place of HiDs. Finally, we examined whether cortical amyloid deposition was associated with degeneration of hippocampal subfields, or moderated the relationships between subfield HiDs and cognition.

Euclidean distance matrices were computed using the scikit-learn package in Python (Pedregosa et al., 2011). We report all *p*-values adjusted for multiple comparisons correction. Multiple comparisons were corrected by controlling the false discovery rate (FDR) using the Benjamini-Hochberg method with the *p.adjust* function from the *stats* package as implemented in R (Benjamini and Hochberg, 1995).

## 3. Results

### 3.1. Neuropsychological assessments and demographics

Forty-six participants were included in all statistical analyses (ADS, 29; Controls, 17). Demographic information for the final sample is provided in Table 1. Also included in Table 1 are inferential statistics for group comparisons of baseline demographics, however strong interpretations of these inferential significance tests are ill-advised (De Boer et al., 2015).

**Figure 2:**
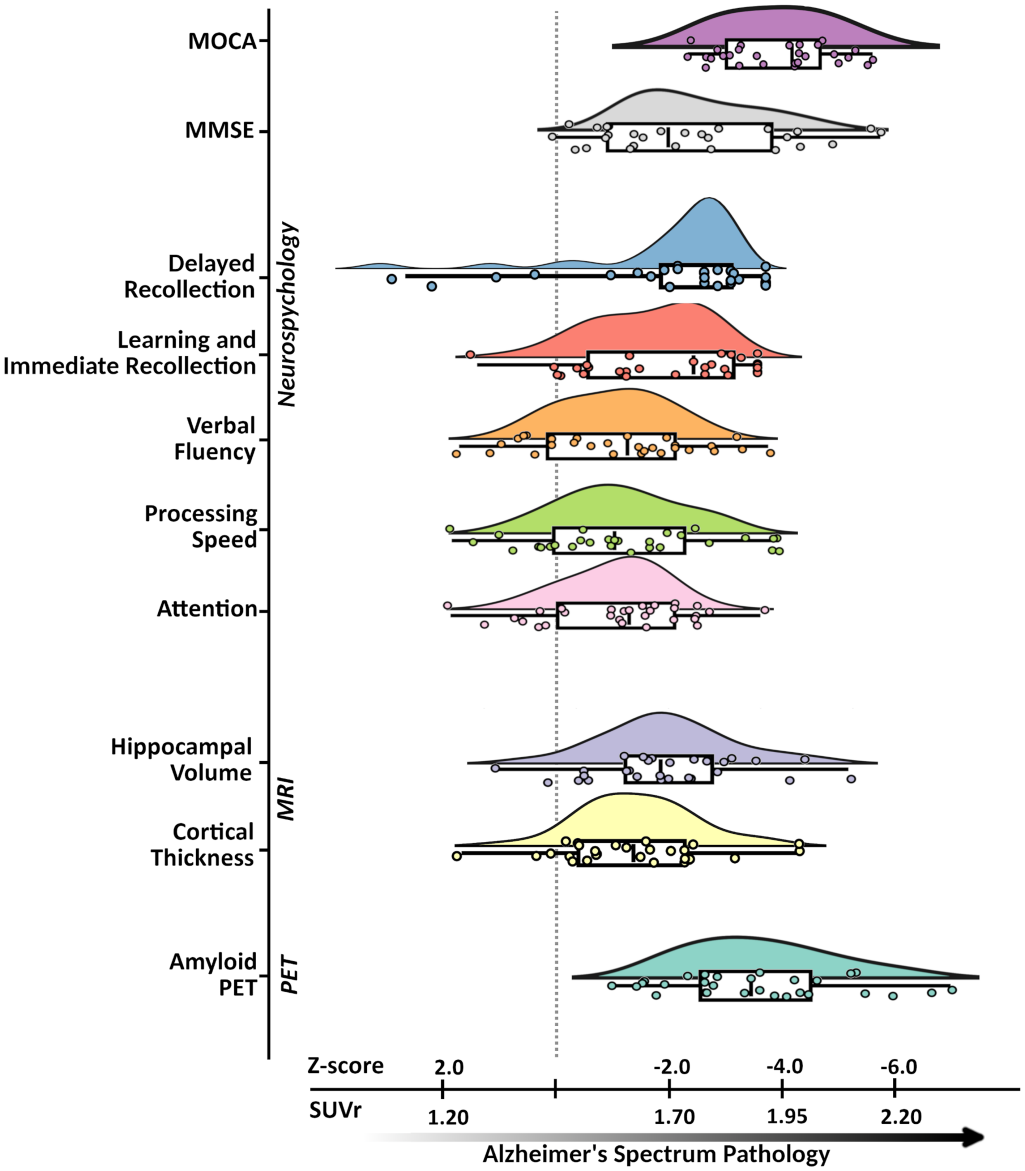
Alzheimer’s disease spectrum sample characteristics. An illustration of the neuropsychological, neuroanatomical, and proteinopathy characteristics of the Alzheimer’s disease spectrum group. Subplots from top to bottom represent pathological deviations along the x-axis (in SUVr for amyloid-PET and z-scores for all other metrics) with respective labels along the y-axis: MoCA, MMSE, Immediate Recollection, Delayed Recollection, Verbal Fluency, Processing Speed, Attention, Hippocampal Volume, Cortical Thickness, Amyloid-β PET. Subplots were all generated from ADS group data where each point represents a given participants’ standardized score based on the control group. Descriptive features of each plot include density, and cumulative probability values for the quantiles with the median (box plot: 25, 50, 75%), and upper and lower limits (whiskers: 2, 98%). MoCA, Montreal Cognitive Assessment; MMSE, Mini-Mental State Exam; PET, positron emission tomography; MRI, magnetic resonance imaging; SUVr, standardized uptake value ratios.

The groups were matched on age, sex, and education. Clinical assessments (e.g., MMSE, MOCA), cortical thickness, and whole hippocampal volume data of our ADS group were normalized to the mean and standard deviation of comparable data from the cognitively normal control group and were paired with the neuropsychological data. By computing z-scored deviations from healthy participants of these measures for all participants in the ADS group, we can visualize changes in different neuropsychological and neuroanatomical measures together in the same plot in parallel with amyloid deposition (Figure 2).

Here, subplots shifted further to the right represent measures which deviate more from controls. Participants on the ADS performed significantly worse than controls across all cognitive domains (Wiesman et al., 2021, 2022). Statistical results for all neuropsychological measures are reported in Table 1, and comparative sample distributions of controls and ADS groups for all neuropsychological data are shown in Supplementary Figure S1.

### 3.2. Group differences in whole hippocampal volumes

We tested for differences in whole hippocampal volumes between individuals on the ADS and healthy control participants. These models revealed significant group differences in left (*F*(1,44) = 34.98, 95% CI [−1015.58, −499.13], *p*_adj_ < 0.001) and right (F(1,44) = 17.49, 95% CI [−836.02, −292.14], *p*_adj_ < 0.001) hippocampus, such that individuals on the ADS exhibited reduced whole hippocampal volumes relative to controls. We then conducted a post-hoc test to examine whether the observed effects in whole hippocampal volume differed between ADS subgroups (i.e., AD vs. MCI). No significant differences were observed between ADS subgroups in either right or left whole hippocampus volumes (Supplementary Table S3).

### 3.3. Group differences in subfield-specific hippocampal volumes

**Figure 3:**
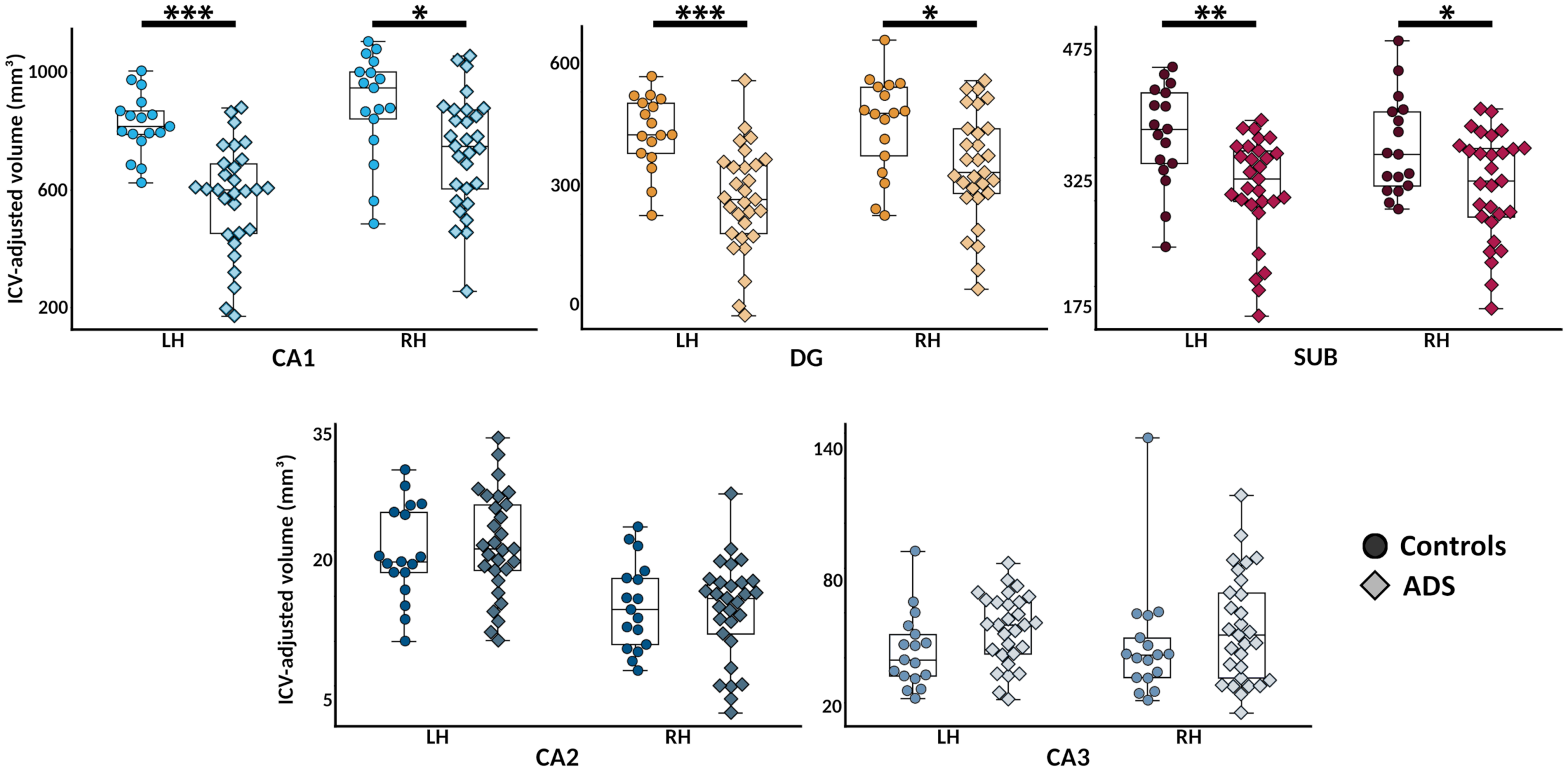
Hippocampal subfield atrophy in participants on the Alzheimer’s disease spectrum. Bilateral hippocampal subfield volumes (in mm3; adjusted for total intracranial volume [ICV]) are plotted for each group for visualization. Color indicates different subfields, and shades and point shapes are doubly indicative of group (darker and circle = controls, lighter and diamond = ADS). In each boxplot, hemisphere is denoted on the x-axis, and subfield volumes are indicated on the y-axis. *pFDR < 0.05, **pFDR < 0.01, ***pFDR < 0.001. CA, cornu ammonis; DG, dentate gyrus; SUB, subiculum; ADS, Alzheimer’s Disease Spectrum.

To evaluate the degeneration of hippocampal subfields among individuals on the ADS, we computed ANCOVAs for group differences in each of the five subfields bilaterally (e.g., left and right CA1, CA2, CA3, dentate gyrus, and subiculum). As shown in Figure 3, these models revealed significant group differences in left (F(1,44) = 33.29, 95% CI [−378.89, −182.64], *p*_adj_ < 0.001) and right (F(1,44) = 7.40, 95% CI [−273.99, −40.82], *p*_adj_ = 0.018) CA1, left (*F*(1,43) = 26.27, 95% CI [−256.09, −111.47], *p*_adj_ < 0.001) and right (F(1,44) = 6.56, 95% CI [−182.81, −21.79], *p*_adj_ = 0.023) dentate gyrus, and left (F(1,43) = 14.90, 95% CI [−109.34, −34.30], *p*_adj_ = 0.001) and right (F(1,43) = 10.34, 95% CI [−94.15, −21.58], *p*_adj_ = 0.006) subiculum. In bilateral CA1, dentate gyrus, and subiculum, these differences were such that individuals on the ADS exhibited reduced hippocampal volumes relative to controls. No group differences were observed in either left (F(1,44) = 0.33, 95% CI [−2.49, 4.49], *p*_adj_ = 0.630) or right (F(1,44) = 0.07, 95% CI [−3.75, 2.85], *p*_adj_ = 0.782) CA2, nor in left (F(1,44) = 3.84, 95% CI [−0.27, 19.46], *p*_adj_ = 0.080) or right (F(1,44) = 0.85, 95% CI [−8.40, 22.57], *p*_adj_ = 0.327) CA3. We then conducted a series of post-hoc tests to examine whether the observed effects in the CA1, dentate gyrus, and subiculum subfields differed between ADS subgroups (i.e., AD vs. MCI). No significant differences were observed between ADS subgroups (Supplementary Table S4). We would like to note that these exploratory subgroup analyses may have been limited by reduced statistical power as a result of dividing the ADS sample into small subgroups, and should be interpreted with caution. Subsequent subfield analyses were limited to subfields which showed significant group differences in the initial group difference ANCOVA models (i.e., CA1, dentate gyrus, and subiculum).

### 3.4. ADS profiles of hippocampal subfield atrophy relate to cognitive impairment

We examined the association between cognitive impairments and the observed degeneration of hippocampal subfields in participants on the ADS. Individual differences in hippocampal degeneration were first computed across all subfields per each member of the ADS group, by deriving the mean Euclidean distance between each patient’s vectorized subfield volumes and those of all healthy controls, resulting in hippocampal degeneration scores (HiDs). These HiDs were then included in separate partial correlations with MMSE, MoCA, and immediate and delayed recollection scores. Significant associations were found between reduced MMSE scores and increased HiDs (*r* = −0.55, 95% CI [−0.89, −0.20], *p*_adj_ = 0.013), but not between MoCA scores and HiDs (*r* = −0.24, 95% CI [−0.62, 0.14], *p*_adj_ = 0.210). Further, significant associations were found between reduced immediate (*r* = −0.42, 95% CI [−0.78, −0.05], *p*_adj_ = 0.047) and delayed recollection (*r* = −0.36, 95% CI [−0.73, 0.00], *p*_adj_ = 0.047) scores and increased HiDs.

**Figure 4:**
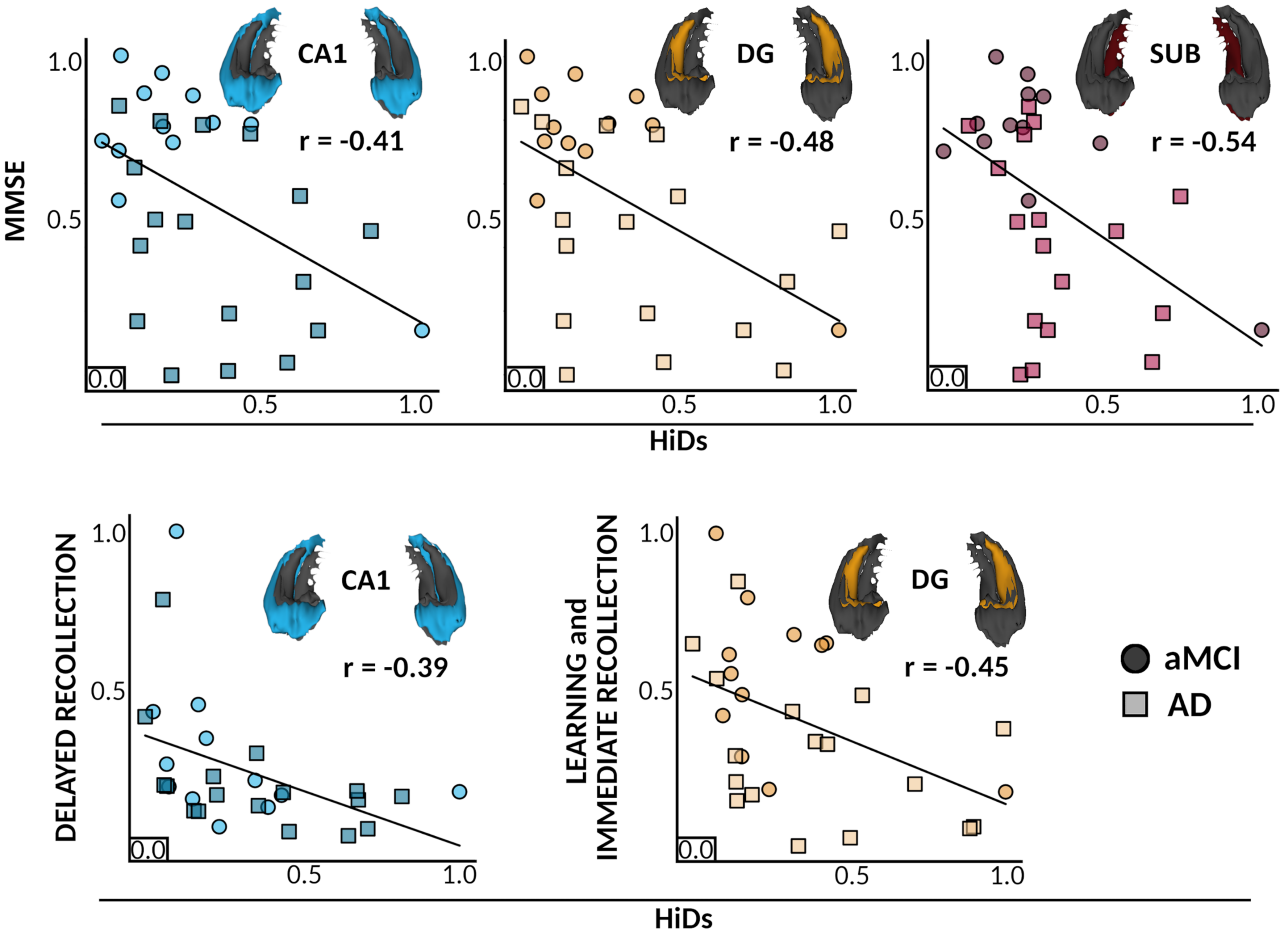
Individual differences in Hippocampal subfield atrophy relate to distinct cognitive deficits. Colors and inlaid 3D subfield depictions indicate different hippocampal subfields for each plot. Colors indicate different subfields, and shades and point shapes are doubly indicative of ADS subgroup (circle = aMCI, squares = AD), though note that statistics are based on the model with all ADS participants combined. In each boxplot, scaled HiDs are denoted on the x-axis, and scaled performance scores of neuropsychological tests (MMSE, Immediate Recollection, Delayed Recollection) are shown on the y-axis. Partial correlations are shown between HiDs and general cognitive status as measured by the MMSE (top), and HiDs and domain-specific cognitive scores for learning and memory (bottom). All associations were significant at a corrected pFDR <0.05. CA, cornu ammonis; DG, dentate gyrus; SUB, subiculum; MMSE, mini-mental state exam; HiDs, Hippocampal Degeneration score; ADS, Alzheimer’s Disease Spectrum; aMCI, amnestic Mild Cognitive Impairment.

To test whether these associations between HiDs and cognitive measures were unique to degeneration of specific subfields, we recomputed HiD scores per each subfield separately using left and right hemispheric values for each member of the ADS group. Here, we focused on those cognitive measures that showed significant associations with the whole-hippocampus HiDs (e.g., MMSE, and immediate and delayed recollection). We found significant associations between reduced immediate recollection performance and increased dentate gyrus HiDs (*r* = −0.45, 95% CI [−0.80, −0.10], *p*_adj_ = 0.042), and between reduced delayed recollection performance and increased CA1 HiDs (*r* = −0.39, 95% CI [−0.76, −0.03], *p*_adj_ = 0.045; Figure 4). Further, significant associations were found between reduced MMSE scores and increased HiDs from CA1 (*r* = −0.41, 95% CI [−0.77, −0.05], *p*_adj_ = 0.045), the dentate gyrus (*r* = −0.48, 95% CI [−0.83, −0.14], *p*_adj_ = 0.036), and the subiculum (*r* = −0.54, 95% CI [−0.91, −0.16], *p*_adj_ = 0.036; Figure 4). There was a trending association observed between increased CA1 HiDs and reduced immediate recollection performance (*r* = −0.39, 95% CI [−0.75, 0.02], *p*_adj_ = 0.053). No significant associations were found between dentate gyrus HiDs and delayed recollection performance (r = −0.38, 95% CI [−0.74, −0.01], *p*_adj_ = 0.114), nor between the subiculum and immediate (*r* = −0.34, 95% CI [−0.74, 0.06], *p*_adj_ = 0.115) or delayed (*r* = −0.218, 95% CI [−0.57, 0.20], *p*_adj_ = 0.347) recollection performance.

Next, we conducted a series of exploratory regression models to determine whether the reported effects of HiDs on cognitive measures significantly differed by ADS subgroup level (i.e., AD vs. MCI). We found no evidence for any such moderation effect (Supplementary Table S5). Note, exploratory analyses may have been limited by reduced statistical power as a result of dividing the ADS sample into subgroups. To test the possibility that these associations between degeneration of subfields and cognition could be due to non-hippocampus-specific cortical atrophy, we computed these subfield HiDs-cognition models substituting global cortical thickness estimates from FreeSurfer for subfield HiDs. None of the reported associations with cognitive scores were found when substituting global cortical thickness for HiDs (Supplementary Table S6), indicating a degree of hippocampal specificity for these effects.

### 3.5. Associations with cortical deposition of amyloid-β

We also examined effects of cortical amyloid deposition on the degeneration of hippocampal subfields and their relationships to cognition. We found that the amount of global cortical amyloid-β, measured across a set of amyloid-accumulating cortical areas, did not significantly relate to any subfield HiDs (CA1: *r* = 0.14, 95% CI [−0.25, 0.53], *p* = 0.882; dentate gyrus: *r* = 0.24, 95% CI [−0.14, 0.62], *p* = 0.272; subiculum: *r* = 0.16, 95% CI [−0.24, 0.55], *p* = 0.706; Supplementary Figure S2), nor did it moderate any of the previously-reported effects of hippocampal degeneration on cognitive measures (MMSE~CA1: *β* = 0.16, 95% CI [−0.19, 0.52], *p* = 0.292; MMSE~dentate gyrus: *β* = 0.14, 95% CI [−0.23, 0.51], *p* = 0.418; MMSE~subiculum: *β* = 0.12, 95% CI [−0.23, 0.44], *p* = 0.507; immediate recollection~dentate gyrus: *β* = 0.11, 95% CI [−0.24, 0.46], *p* = 0.517; delayed recollection~CA1: *β* = 0.27, 95% CI [−0.08, 0.61], *p* = 0.173; Supplementary Figure S3).

## 4. Discussion

We used high-resolution *in-vivo* imaging of human hippocampal subfield volumes and detailed neuropsychological testing of learning and memory abilities to examine the impact of subfield-specific hippocampal degeneration on cognitive function in participants on the ADS. Our results replicate previous findings showing that hippocampal subfields among individuals on the ADS are significantly atrophied as compared to healthy controls (Figure 3). The pattern of subfield atrophy was such that volumes of participants on the ADS were decreased in CA1, dentate gyrus, and subiculum, a pattern of atrophy previously reported with individual subfield component contributions (Apostolova et al., 2006; Mueller et al., 2010; La Joie et al., 2013; Wisse et al., 2014; Yushkevich et al., 2015; Kälin et al., 2017; Wolk et al., 2017). To next examine the associations between individualized degeneration scores and cognition, we utilized a mean Euclidean distance approach to quantify the bilateral degeneration of each subfield in each individual on the ADS relative to all controls. This approach allowed for the quantification of individual-level subfield degeneration across hemispheres relative to all control participants, giving a more nuanced measure of degeneration than the raw volumetrics. Extending previous findings, we show that the degree of degeneration of hippocampal subfields is reflected in neurocognitive deficits in participants with ADS pathology (Figure 4). Greater subfield decrements relative to healthy controls were significantly linked to reduced cognitive performance. Specifically, we found significant associations between bilateral atrophy in CA1 and dentate gyrus and performance on tests of immediate recollection, delayed recollection, and general cognitive status (i.e., MMSE scores), such that greater AD-related atrophy was associated with worse cognitive performance. In addition, degeneration scores derived from the subiculum were significantly associated with lower scores on the MMSE.

Atrophy of the hippocampal subiculum has been widely reported in both healthy aging and participants on the ADS (Apostolova et al., 2006, 2010; Hanseeuw et al., 2011; La Joie et al., 2013; Wisse et al., 2014; Khan W. et al., 2015; Wolk et al., 2017; Huang et al., 2020; Izzo et al., 2020). Interestingly, others have reported that atrophy of the subiculum is specific to participants on the ADS, such that only amyloid-positive participants with cognitive impairments showed significant subiculum atrophy (La Joie et al., 2013). Recent studies have also shown that atrophy of the subiculum is significantly associated to neuropsychological test scores (Carlesimo et al., 2015; Ogawa et al., 2019; Zhao et al., 2019). The current study supports both positions, as declines in subiculum volume were found in participants on the ADS relative to healthy controls, and these decrements were related to lower MMSE scores. To our knowledge, only one other study has found a similar linear association between subiculum volumes in participants on the ADS and a measure of general cognitive status (i.e., MoCA; Ogawa et al., 2019). This may be due, in part, to the fact that the subiculum is a relatively understudied subfield. Although other studies have previously reported decreased volume in the subiculum in patients with AD, few have connected such decreases with cognitive outcomes. These results suggest that a nuanced approach to examining associations between hippocampal subfield degeneration and cognition is important to fully understand the effects of AD on brain structure.

In addition to our subiculum findings, we show that subfield volumes are reduced in CA1 and dentate gyrus in participants on the ADS relative to controls. These patterns of atrophy were related to learning and memory abilities and to general cognitive status. A myriad of studies have reported atrophy of the CA1 and dentate gyrus in normal aging (Mueller et al., 2010; Apostolova et al., 2012; Wisse et al., 2014; Kurth et al., 2017; Malykhin et al., 2017; Parker et al., 2019), which is accelerated in participants on the ADS (Apostolova et al., 2006, 2010; Mueller et al., 2010; La Joie et al., 2013; Wisse et al., 2014; Khan W. et al., 2015; Yushkevich et al., 2015; Kälin et al., 2017; Carlson et al., 2020; Huang et al., 2020). Reductions in CA1 volume have been associated with decreases in declarative memory and semantic encoding (Atienza et al., 2011), declarative encoding and retrieval (Fouquet et al., 2012; Ogawa et al., 2019), as well as prospective memory (Nurdal et al., 2020), while dentate gyrus atrophy has been implicated in worsening performance on pattern separation tasks (Lee et al., 2020; Riphagen et al., 2020), assessments that examine learning and memory such as the WMS (Ogawa et al., 2019), and clinical screens, including the MMSE and MoCA (Ogawa et al., 2019). This previous work implies far-reaching cognitive consequences of AD-related degeneration of these structures, which our findings also support. Importantly, of these studies, those which examined associations between these behavioral measures and volume changes in various subfields in the context of AD did not impose inclusion criteria based on amyloid status. To our knowledge, this is the first study to use field-standard imaging acquisition for hippocampal subfield parcellation in combination with biomarker-definition of both the healthy control and patient groups, representing a valuable replication of previous work on AD-related subfield decrements and their relevance to cognitive decline.

The current study did not find significant group differences in either the CA2 or CA3 hippocampal subfield volumes, which is consistent with prior studies that have parceled these subfields (Apostolova et al., 2006; Wisse et al., 2014). However, other studies have found group differences in these subfields, particularly at later stages of the disease (Apostolova et al., 2010; Mueller et al., 2010; Kälin et al., 2017). It is possible that the CA2 and CA3 subfields may be spared well into the disease process (Schönheit et al., 2004; Mueller et al., 2008; Apostolova et al., 2010), or that CA3 follows a nonlinear trajectory, with transient increases at earlier stages and decreases found later (McKeever et al., 2020). It is also possible that as the smallest subfields, CA2 and CA3 may be particularly impacted by subtle errors in segmentation (Mueller et al., 2008, 2010). One common approach to manage this concern is to combine the CA2 and CA3 subfields with other neighboring volumes, but this procedure is highly inconsistent in the literature: some groups combine CA2 with CA3 (Hanseeuw et al., 2011), others combine CA2 with CA1 and CA3 with dentate gyrus (Mueller et al., 2010), and others combine both CA2 and CA3 with dentate gyrus (Carlson et al., 2020). As such, in the present study, we elected to retain each independent subfield estimate to increase reproducibility by reducing the number of arbitrary post-processing steps.

This study serves as an essential replication of previous work, while also adding to existing knowledge in several ways. This is one of the first studies relating hippocampal subfield volumes and cognitive abilities in which group assignments for both ADS and healthy controls were determined based on amyloid status. This is likely due to the high difficulty and cost in obtaining such markers, especially in the absence of clinical symptoms (cognitively normal controls). However, biomarker confirmation of ADS/control groups is essential to confirm that early cognitive declines experienced by participants with MCI are indeed the result of AD pathology, aligning with the recent emphasis on using biological definitions of AD for research (Jack et al., 2018). Our neuroimaging approach follows field-standard recommendations for state-of-the-art imaging and acquisition of high-resolution structural scans for optimal parcellation of hippocampal subfields. In particular, recent studies have highlighted two important considerations in the context of our methods and research questions. Special focus has been given to the development of sub-millimeter imaging sequences to investigate hippocampal subfields more precisely, as standard T1-weighted MRI scans have been shown to be less reliable than studies which introduce a second higher resolution scan into the processing workflow (de Flores et al., 2015; Yushkevich et al., 2015; Olsen et al., 2019; Wisse et al., 2021). The present study took the latter approach and used both T1- and T2-weighted scans, which is optimal for examining hippocampal subfields. Moreover, Mueller et al. (2018) have directly compared the differential performance of these approaches in the context of MCI, showing significant improvements in separation of healthy and MCI groups when subfield measures are derived from dedicated high-resolution images. Finally, by quantifying the degeneration (i.e., HiDs, Figure 1) of each subfield in each individual on the ADS using a multidimensional approach across hemispheres and relative to a sample of cognitively normal, amyloid-negative individuals, we were able to show how AD-related decrements in each subfield reflect appreciable changes on cognitive metrics. This approach allowed for us to compute the degeneration of each subfield at the individual level, giving a more fine-grained view of decrements than the raw volumetric measurements alone by incorporating the associated asymmetry of hippocampal volumes across hemispheres without smoothing this meaningful variability (Maruszak and Thuret, 2014; Sarica et al., 2018; Ogawa et al., 2019; Zhao et al., 2019; Huang et al., 2020), while minimizing the number of comparisons necessary to maintain statistical sensitivity.

While our study measured several key disease metrics and controlled for many important variables, some limitations still exist. The current study did not find significant effects of cortical amyloid deposition on the reported associations between subfield HiDs and cognition (Supplementary Figure S3). This is consistent with prior studies (Schönheit et al., 2004; La Joie et al., 2012), and while it was important to include an amyloid biomarker for group distinctions, this also highlights a limitation and direction for future research. That is, the current study did not measure tau deposition, which has been previously shown to relate to patterns of cortical neurodegeneration and declines on cognitive measures (Hansson et al., 2017; Jack et al., 2018; Tardif et al., 2018; Maass et al., 2019; McCollum et al., 2021; Mehta and Schneider, 2021; Therneau et al., 2021). Future studies should include measures of tau burden to further inform the atrophic patterns and associations to cognition observed here. The current study also did not examine AD-related genetic factors (Mueller et al., 2008; Schuff et al., 2008; O’Dwyer et al., 2012; Tardif et al., 2018; McKeever et al., 2020; Murray et al., 2021). Hippocampal subfields have been linked previously to genetic contributions (van der Meer et al., 2020), making this an important next step for our work. In this study we also focused our analyses on hippocampal subfields volumes. While the computation of volume measures is most common for investigations of large-scale neurodegeneration, future research should also consider shape morphometry: a complementary approach that has been shown to provide novel insights into morphology and cognition (Voineskos et al., 2015; Valdés Hernández et al., 2017). Although the hippocampus is a key region in the pathological process of AD, it serves as a hub for several functional networks and is highly connected with other regions in the brain (Ranganath and Ritchey, 2012). Therefore, future studies should investigate whether structural and/or functional changes in hippocampal-connected cortical regions play a mediating role in our observed associations.

In conclusion, our results reinforce previously reported atrophy in hippocampal subfield volumes, and the significant role these pathological changes play in cognitive impairment among participants on the Alzheimer’s Disease Spectrum. Subfield-specific atrophy in the human hippocampus may constitute a sensitive measure of neuropathology in ADS groups, and future studies of the functional, structural, and molecular mediators of the neurocognitive relationships reported herein are warranted.

## Data availability statement

The raw data supporting the conclusions of this article will be made available by the authors, without undue reservation.

## Ethics statement

The studies involving humans were approved by Institutional Review Board at the University of Nebraska Medical Center. The studies were conducted in accordance with the local legislation and institutional requirements. Written informed consent for participation in this study was provided by the was provided by the participants themselves, or in the case where capacity to consent was questionable, by their legally-authorized representative.

## Author contributions

NCH: conceptualization, data curation, formal analysis, methodology, software, visualization, writing – original draft. CE: conceptualization, formal analysis, methodology, supervision, writing – original draft and editing. AW and TW: conceptualization, investigation, methodology, project administration, resources, funding acquisition, validation, supervision, writing – review and editing. PM: resources, data curation, writing – review and editing. MS: investigation, data curation, writing – review and editing. CJ: investigation, formal analysis, data curation, writing – review and editing. SW: resources, investigation, writing – review and editing. DM: conceptualization, investigation, methodology, resources, funding acquisition, validation, supervision, writing – review and editing. All authors contributed to the article and approved the submitted version.
