# Supplementary material for: Piecing it together: atrophy profiles of hippocampal subfields relate to cognitive impairment along the Alzheimer’s disease spectrum
Source: Front Aging Neurosci. 2023 Oct 31;15:1212197. doi: 10.3389/fnagi.2023.1212197 (PMC10644116; doi:10.3389/fnagi.2023.1212197)
Supplement: Supplementary file 1 [file Table_1.DOCX]

Supplementary Material

Piecing it together: atrophy profiles of hippocampal subfields relate to cognitive impairment along the Alzheimer’s disease spectrum

Nicholas J. Christopher-Hayes^1,2†^, Christine M. Embury^1,3†^, Alex I. Wiesman^4*^, Pamela E. May^5^, Mikki Schantell^1,6^, Craig M. Johnson^7^, Sara L. Wolfson^8^, Daniel L. Murman^5,9^, Tony W. Wilson^1,6,10^

*** Correspondence:**Alex I. Wiesman, PhD

Montreal Neurological Institute

McGill University

Montreal, Quebec, Canada

[alexander.wiesman@mcgill.ca](mailto:alexander.wiesman@mcgill.ca)

# Supplementary Figures and Tables

## Supplementary Tables

| **Table S1. No significant differences in demographics or general cognitive status based on MRI data exclusions.** | | | |
| --- | --- | --- | --- |
|  | ***estimate*** | ***z-value*** | ***p-value*** |
| Age (yrs) | 0.02 | 0.41 | 0.68 |
| Group (HC) | 0.99 | 0.83 | 0.41 |
| Sex (female) | 0.16 | 0.21 | 0.83 |
| Education | -0.29 | -1.91 | 0.05 |
| MoCA | -0.14 | -0.88 | 0.37 |
| MMSE | 0.14 | 0.85 | 0.39 |
|  | | | |
| Logistic regression with MRI data inclusion/exclusion status as binary outcome. HC = Healthy Control; MOCA = Montral Cognitive Assessment; MMSE = mini-mental state exam. | | | |

| **Table S2. Specifications for models including covariates and/or requiring non-parametric statistics**. | | | |
| --- | --- | --- | --- |
| ***y*** | ***x1*** | ***covariates (x2:xn)*** | ***model type*** |
| *Neuropsychological tests (manuscript section 3.1)* | | | |
| Delayed Recollection | Group(HC/ADS) |  | non-parametric (Wilcoxon) |
| *Group difference tests (manuscript sections 3.2-3.3)* | | | |
| Whole Hippocampus  (right & left) | Group(HC/ADS) | Age | parametric |
| Whole Hippocampus  (right & left) | Group(aMCI/AD) | Age | parametric |
| CA3(right) | Group(HC/ADS) |  | non-parametric |
| CA1(left) | Group(HC/ADS) | Age | parametric |
| CA1(left) | Group(aMCI/AD) | Age | parametric |
| SUB(right & left) | Group(HC/ADS) | Age | parametric |
| SUB(right & left) | Group(aMCI/AD) | Age, Sex, Education | parametric |
| *HiDs effects on Cognition (manuscript section 3.4)* | | | |
| MMSE | HiDs | Age | parametric |
| Immediate Recollection | HiDs | Age | parametric |
| Delayed Recollection | HiDs |  | non-parametric |
| MMSE | CA1 HiDs |  | non-parametric |
| Immediate Recollection | CA1 HiDs | Age | parametric |
| Immediate Recollection | CA1 HiDs x Group(aMCI/AD) | Age | parametric |
| Delayed Recollection | CA1 HiDs |  | non-parametric |
| Delayed Recollection | CA1 HiDs x Group(aMCI/AD) |  | non-parametric |
| Immediate Recollection | DG HiDs | Age | parametric |
| Immediate Recollection | DG HiDs x Group(aMCI/AD) | Age | parametric |
| Delayed Recollection | DG HiDs x Group(aMCI/AD) |  | non-parametric |
| Delayed Recollection | DG HiDs |  | non-parametric |
| MMSE | SUB HiDs | Age | parametric |
| Immediate Recollection | SUB HiDs | Age | parametric |
| Immediate Recollection | SUB HiDs x Group(aMCI/AD) | Age | parametric |
| Delayed Recollection | SUB HiDs |  | non-parametric |
| Delayed Recollection | SUB HiDs x Group(aMCI/AD) |  | non-parametric |
| Immediate Recollection | Global Cortical Thickness | Age | parametric |
| *Associations of SUVr, HiDs, and interactions on Cognition (manuscript section 3.5)* | | | |
| CA1 HiDs | SUVr |  | non-parametric |
| DG HiDs | SUVr |  | non-parametric |
| SUB HiDs | SUVr |  | non-parametric |
| MMSE | CA1 HiDs x SUVr |  | non-parametric |
| Delayed Recollection | CA1 HiDs x SUVr |  | non-parametric |
| MMSE | DG HiDs x SUVr |  | parametric |
| Immediate Recollection | DG HiDs x SUVr | Age | parametric |
| MMSE | SUB HiDs x SUVr | Age | parametric |
| All non-parametric models used permutation testing unless otherwise noted.  HC = Healthy Control; aMCI = amnestic Mild Cognitive Impairment, ADS = Alzheimer’s Disease Spectrum.  MOCA = Montral Cognitive Assessment; MMSE = mini-mental state exam.  CA = cornu ammonis, DG = dentate gyrus, SUB = subiculum.  HiDs = Hippocampal Degeneration Score. | | | |

| **Table S3. ADS subgroup differences in whole hippocampal volumes**. | | | | | |
| --- | --- | --- | --- | --- | --- |
|  | ***df*** | ***F-value*** | ***2.5%*** | ***97.5%*** | ***p-value*** |
| *Whole Hippocampus* | | | | | |
| *Left* |  |  |  |  |  |
| Group (aMCI/AD) | 1, 26 | 0.59 | -509.01 | 231.94 | 0.45 |
| *Right* |  |  |  |  |  |
| Group (aMCI/AD) | 1, 26 | 1.01 | -595.93 | -204.83 | 0.32 |
|  | | | | | |
| All hypothesis tests used ANCOVA’s with type II SS. aMCI = amnestic Mild Cognitive Impairment, ADS = Alzheimer’s Disease Spectrum. | | | | | |

| **Table S4. ADS subgroup differences in hippocampal subfield volumes**. | | | | | |
| --- | --- | --- | --- | --- | --- |
|  | ***df*** | ***F-value*** | ***2.5%*** | ***97.5%*** | ***p-value*** |
| *CA1* | | | | | |
| *Left* |  | | | | |
| Group (aMCI/AD) | 1, 26 | 0.52 | -198.79 | 95.79 | 0.48 |
| *Right* |  | | | | |
| Group (aMCI/AD) | 1, 27 | 0.36 | -199.85 | 108.89 | 0.55 |
| *DG* | | | | | |
| *Left* |  | | | | |
| Group (aMCI/AD) | 1, 27 | 0.09 | -119.82 | 88.21 | 0.76 |
| *Right* |  |  |  |  |  |
| Group (aMCI/AD) | 1, 27 | 0.26 | -135.82 | 82.07 | 0.26 |
| *SUB* | | | | | |
| *Left* |  |  |  |  |  |
| Group (aMCI/AD) | 1, 25 | 0.00 | -47.28 | 49.80 | 0.96 |
| *Right* |  |  |  |  |  |
| Group (aMCI/AD) | 1, 25 | 0.01 | -44.02 | 47.73 | 0.93 |
|  | | | | | |
| All hypothesis tests used ANCOVA’s with type II SS. aMCI = amnestic Mild Cognitive Impairment, ADS = Alzheimer’s Disease Spectrum; CA = cornu ammonis, DG = dentate gyrus, SUB = subiculum.  *p < 0.05 | | | | | |

| **Table S5. ADS subgroup moderation of associations between hippocampal subfield atrophy and cognitive outcomes.** | | | | | |
| --- | --- | --- | --- | --- | --- |
|  | ***df*** | ***β*** | ***2.5%*** | ***97.5%*** | ***p-value*** |
| *MMSE* | | | | | |
| *CA1 x*  *Group (aMCI/AD)* | 3, 25 | -0.07 | -0.44 | 0.29 | 0.486 |
|  |  |  |  |  |  |
| *DG x*  *Group (aMCI/AD)* | 3, 25 | 0.19 | -0.44 | 0.82 | 0.536 |
|  |  |  |  |  |  |
| *SUB x*  *Group (aMCI/AD)* | 3, 25 | 0.31 | -0.29 | 0.91 | 0.297 |
|  |  |  |  |  |  |
| *LEARNING* | | | | | |
| DG x  Group (aMCI/AD) | 4, 24 | 0.25 | -0.41 | 0.91 | 0.443 |
|  |  |  |  |  |  |
| *MEMORY* | | | | | |
| *CA1 x*  *Group (aMCI/AD)* | 3, 25 | 0.26 | -0.44 | 0.29 | 0.233 |
|  | | | | | |
| aMCI = amnestic Mild Cognitive Impairment, ADS = Alzheimer’s Disease Spectrum; CA = cornu ammonis, DG = dentate gyrus, SUB = subiculum. | | | | | |

| **Table S6. Associations between global cortical thickness and cognitive outcomes.** | | | | | |
| --- | --- | --- | --- | --- | --- |
|  | ***Df*** | ***β*** | ***2.5%*** | ***97.5%*** | ***p-value*** |
| *MMSE* | | | | | |
| *Global Cortical Thickness* | 1, 27 | 0.95 | -3.07 | 4.96 | 0.633 |
| *Immediate Recollection* | | | | | |
| *Global Cortical Thickness* | 2, 26 | 1.21 | -2.74 | 5.17 | 0.533 |
| *Delayed Recollection* | | | | | |
| *Global Cortical Thickness* | 1, 27 | 0.27 | -3.76 | 4.29 | 0.893 |
|  | | | | | |
| aMCI = amnestic Mild Cognitive Impairment, ADS = Alzheimer’s Disease Spectrum; CA = cornu ammonis, DG = dentate gyrus, SUB = subiculum. | | | | | |

## Supplementary Figures


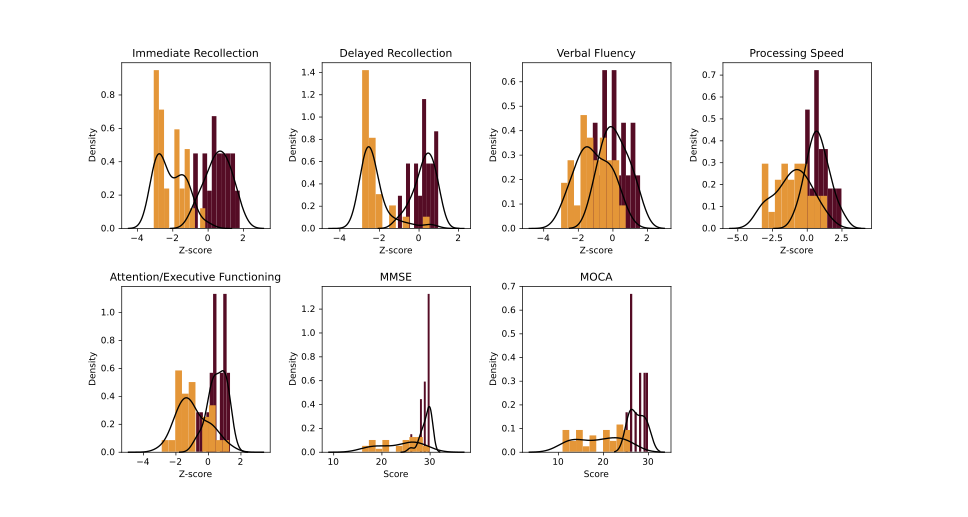


**Figure S1.** **Sample neuropsychological assessment composites and general cognitive status measures.** Probability density of performance distributions for all neuropsychological assessment composites and general cognitive status measures. Colors indicate sample groups in each subplot (yellow = ADS, red = controls). ADS = Alzheimers Disease Spectrum; MOCA = Montral Cognitive Assessment; MMSE = mini-mental state exam.

**
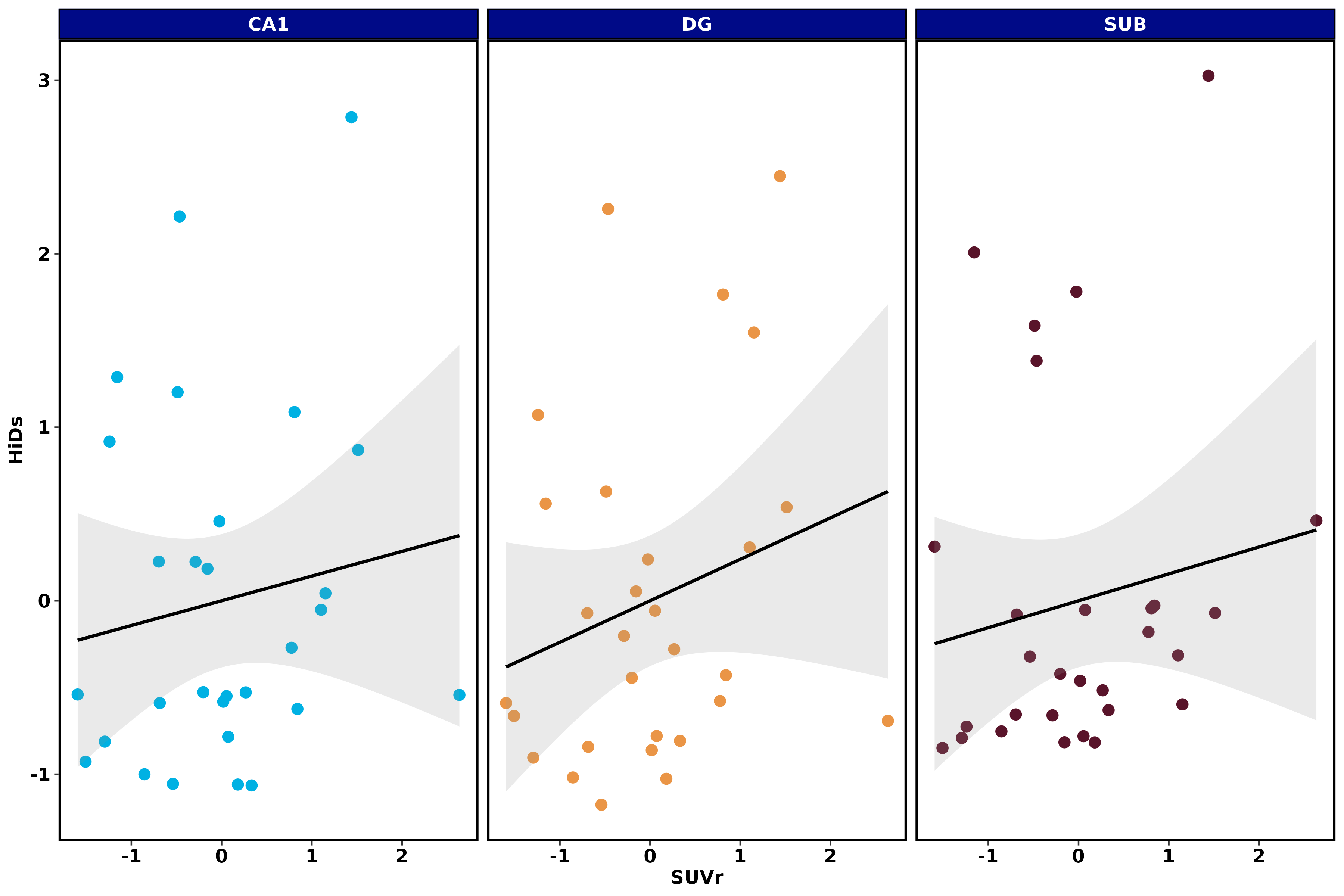
**

**Figure S2.** **Associations between subfield HiDs and cortical deposition of amyloid-β.** Scatterplots represent associations between subfield HiDs and cortical amyloid deposition, with lines-of-best-fit and 95% confidence intervals overlaid. Color indicates different subfields (blue = CA1, yellow = dentate gyrus, red = subiculum). In each boxplot, SUVr is denoted on the x-axis, and HiDs are indicated on the y-axis. SUVr = standardized uptake value ratio; CA = cornu ammonis, DG = dentate gyrus, SUB = subiculum.

**
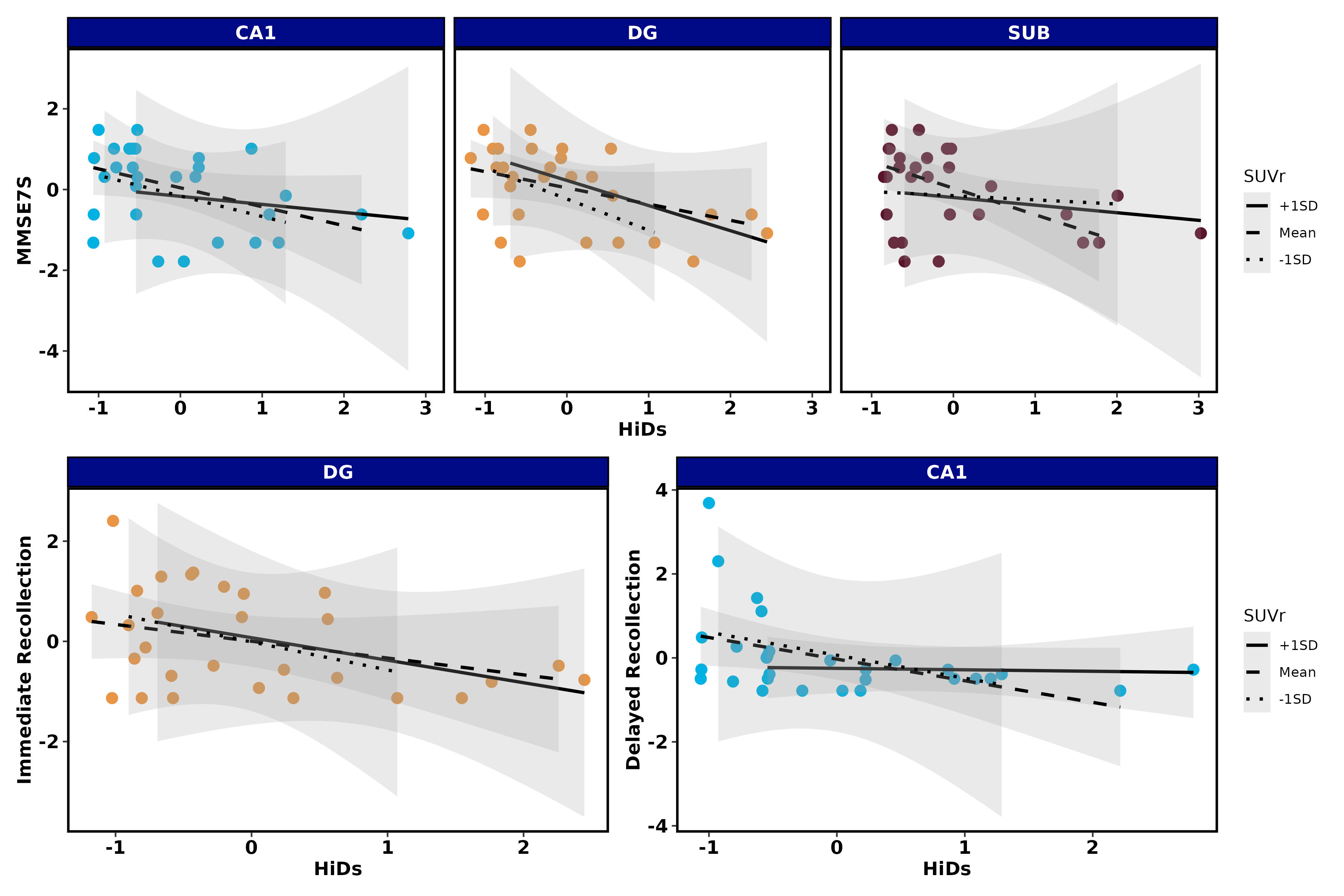
**

**Figure S3.** **Moderation of relationships between HiDs and cognitive measures by cortical deposition of amyloid-β.** Scatterplots represent associations between subfield HiDs and cognitive measures, with lines-of-best-fit and 95% confidence intervals overlaid for each level of cortical amyloid deposition (±1 SD; indicated by different line types). Color indicates different subfields (blue = CA1, yellow = dentate gyrus, red = subiculum). In each boxplot, HiDs are denoted on the x-axis, and neuropsychological tests (MMSE, Immediate Recollection, Delayed Recollection) with scaled performance scores are shown on the y-axis. SUVr = standardized uptake value ratio; CA = cornu ammonis, DG = dentate gyrus, SUB = subiculum, MMSE = mini-mental state exam.
